# The Reign of Law

**Published:** 1897-11

**Authors:** 


### Editorial.

### THE REIGN OF LAW.

THE question of questions at the present time agitating college circles, as well as the dental profession, is that of law in connection with dental education. The existence of State Examining Boards has grown out largely of a jealousy of colleges in general and a selfish desire to prevent overcrowding in the ranks of both dental and medical workers. This is not the reason generally given. On the contrary, the idea is sought to be enforced that these boards are the result of an earnest desire upon the part of many men in the dental and medical professions to uplift college work and make it worthy the more exacting demands of the period. That this is the sole actuating motive of many worthy men cannot be denied; but admitting this, it still leaves an open door for that class to enter who antagonize and have always antagonized college work.

It has been one of the most discouraging features of educational labor on professional lines that those who know the least of this are the ones ever ready to rush into the arena of professional politics for a means to cripple the system, which the few earnest men have sought to build up and make more and more perfect during the passing years.

It is, therefore, with unusual gratification that one man has been found willing to enter the discussion of this subject with a broad conception of the entire matter, and it is hoped that the "Open Letter," by Dr. B. F. Arrington, on another page, will be read carefully by both sides interested in this important subject. It is a clear, logical statement of the questions involved from the altruistic stand-point; and, in the opinion of the writer, meets the issue with a spirit worthy of imitation by those carping critics who assail college work apparently with no other object than the pleasure of antagonizing that which is above their comprehension and to them unattainable.

The examining boards, as at present constituted in the several States, were not organized without considerable opposition from legislative bodies and the public at large; but this gradually succumbed to the pressure until now they exist in all the States of the Union. In the majority they hold a supervising power over the colleges. These, while chartered to confer the right to practise, have had that power rendered a nullity and their diplomas relegated to a value equivalent to an ordinary certificate. There is as yet no method devised to prove the competency of the men who constitute the boards, nor is there any means provided to restrain them from rendering unjust decisions. They are practically a law unto themselves, with no supervising power to control their actions. They are placed in these positions mainly to re-examine graduates from colleges. This presumes that their training is in degree superior to that of those who have given their lives to the work of educating students. If the boards are not thus superior to the teachers in colleges, why are they given these positions of responsibility? Without reflecting on the qualifications of any set of men, it is manifestly unreasonable to suppose that dentists who have been long years in active practice, with no close connections with college work and with no time and little disposition for extended professional culture, should be gifted beyond those who have had opportunities for just the opposite training. It is unreasonable, nay, more, it is absurd, to place men, however good their motives may be, as regulators of the work of men their superiors in professional education. The manager of a large manufacturing establishment would not for a moment think of making a superintendent out of an unskilled artisan and have him judge the product. Yet this is exactly what our laws are doing. Dr. Arrington very forcibly puts this part of the question when he says, "I also honestly believe that dental college faculties, as a whole, are infinitely more competent and better prepared to judge of the qualifications requisite for successful practice and ultimate rendering of good service, to the honor and credit of the profession, than are the majority of dentists who compose dental examining boards."

The other side of this question is that relating to the students, and this Dr. Arrington seems very justly to regard as the most important. The action of some of the State boards in turning down these graduates by wholesale has roused an indignant response from the more intelligent of the dental profession. It is very clear that the college from which these young men graduated could not have been wilfully negligent of their duty, nor could the young men, after their years of study and work, have been incapable of practice in such numbers as have been reported. It is very evident that some State boards have overdone the work and have justly excited the suspicion that they were not as eager to improve the curriculum of colleges as they were to prevent additions to their number in practice. If the latter be the reason, and the evidence is very strong to support it, then the profession should demand the repeal of all laws that permit such a brutal interference with the efforts of young men to engage in an honorable calling.

Whether this has been the case or not, the power exists, and who is there to control it? The decision of these boards is final, and the graduates have no chance of appeal, and having none, they must sacrifice three years of time, labor, and oftentimes money earned through great privations, to the power of a few selfish men, for it is a waste of words to say that such action is based upon a desire to elevate the profession.

The range of subjects entering into the curriculum of dental colleges at the present time stands in no relation to that of a period of twenty years ago. The men who compose the boards of the several States, with scarcely any exception, are incapable of examining on the subjects as now taught. Chemistry, if taught at all, was through lectures and by experiments made by the incumbent of the chair. Bacteriology was an unknown study even five years ago. The study of anatomy was practically confined to the head. Physiology was a run over, as the young men were supposed to require but a superficial knowledge in this direction. Histology, general and special, was not taught in laboratories. If the dental student wished a knowledge of surgery, he had to acquire it outside of the dental college. Even in the supposed essential branches of prothesis and operative dentistry there have been radical advances, and to such an extent that the graduate of a few years back feels as he surveys the field like a door rusting on its hinges, and longs to renew college associations. Dental pathology and materia medica were taught a few years ago in a most perfunctory manner; now the relations to general pathology and therapeutics are made of vital importance. That some schools endeavored to fulfil all these requirements is acknowledged, but they were very exceptional and their work bore no relation to the present standard of requirements. If this statement be true, and all conversant with the facts know it to be true, how can these men undertake to traverse the knowledge gained by the students who have covered this entire work?

The result of all this is that young men are subjected, in many instances, to the examination of men ignorant, first of the proper methods of examination, and equally ignorant of the subjects they are supposed to examine upon and render a verdict for the good or ill of the poor victims of unreasonable law.

It is not to be understood that it is thought all law is undesirable. It is recognized that the statutes of the several States, however imperfectly formed, have had, in degree, a beneficial effect in stirring educators to a more healthful activity, and it would, perhaps, be unwise to repeal all laws upon dentistry and medicine, as has been suggested in certain quarters; but if the present laws were amended, so that no further re-examinations were permitted, and diplomas of one State were to be accepted in every State of the Union, and the suggestions of Dr. Arrington in regard to censors be added, it is thought we might move along for some years in a greater degree of harmony than exists at present.

One thing is very evident, that the National Association of Dental Examiners has passed the stage of tolerance, and if the State boards would cease to send delegates to it, one cause of a vast deal of trouble would be removed. As it stands to-day, it is a positive menace to the colleges of the country, and should be abated without any reservations as an obstruction to healthy dental progress. When it is remembered that this body has as part of its membership men not connected with any State boards, the absurdity of their pretending to control the work of the colleges becomes painfully apparent. When, also, one of these non-representative members is secretary of the Committee on Dental Colleges, the work of this body becomes absolutely farcical. The report of their proceedings would furnish most amusing reading were it not of serious moment to the parties most interested.

The time has arrived when this subject should be met with some decided action, for if colleges and students are to be the victims of an irresponsible national organization, these should know the worst, and the earlier this information is forthcoming the better it will be for all concerned.

There are, unfortunately, a few men of prominence as educators in dentistry, who sit supinely in our conventions, or openly defend all law, however administered. The time is coming, if not already here, when this kind of spirit will not be tolerated. It bodes no good to a profession when ignorance and intolerance commands, and it is equally unfortunate when intelligence succumbs to this power and apologizes or supports it. Let the colleges of this country demand to be considered on this question of State boards, and in order to be heard, let them insist on being represented on every State board, and not, as now, completely excluded. It is an insult to every man connected with colleges to assume that they could not honestly examine students; but this is not surprising, in view of the fact that re-examination practically assumes that every professor is unworthy of confidence, and his decision, as to the standing of a student, not entitled to any consideration.

We have had time for discussion, and it has profited nothing. The time has come when the colleges of the United States must resist to the extreme, if necessary, these encroachments upon their just rights. It is due these educational institutions that at the next meeting of the National Association of Dental Faculties this subject should assume a very large share of attention, and it should be seriously considered whether some means should not be taken to prevent this growing interference with the legal rights of the schools.

